# Psychotropic and other medicine use at time of death by suicide: a population‐level analysis of linked dispensing and forensic toxicology data

**DOI:** 10.5694/mja2.51985

**Published:** 2023-05-25

**Authors:** Kate M Chitty, Nicholas A Buckley, Jessy Lim, Zein Ali, Jennifer L Schumann, Rose Cairns, Benjamin Daniels, Sallie A Pearson, David B Preen, Andrea L Schaffer

**Affiliations:** ^1^ The University of Sydney Sydney NSW; ^2^ The University of Western Australia Perth WA; ^3^ Poisons Information Centre Children's Hospital at Westmead Sydney NSW; ^4^ Victorian Institute of Forensic Medicine Monash University Melbourne VIC; ^5^ Monash Addiction Research Centre Monash University Melbourne VIC; ^6^ Monash University Melbourne VIC; ^7^ The University of New South Wales Sydney NSW; ^8^ The Bennett Institute of Applied Data Science University of Oxford Oxford United Kingdom

**Keywords:** Suicide, Pharmacoepidemiology, Toxicology

## Abstract

**Objectives:**

To determine the numbers and types of medicines dispensed around the time of death to people who die by suicide; to compare the medicines recently dispensed and those recorded in post mortem toxicology reports.

**Design, setting, participants:**

Analysis of linked National Coronial Information System (NCIS) and Pharmaceutical Benefits Scheme (PBS) data from the Australian Suicide Prevention using Health Linked Data (ASHLi) study, a population‐based case series study of closed coronial cases for deaths of people in Australia aged ten years or more during 1 July 2013 – 10 October 2019 deemed by coroners to be the result of intentional self‐harm.

**Main outcome measures:**

Proportions of people to whom medicines were dispensed around the time of death, by medicine group, class, and specific medicine; comparison of medicines recently dispensed and those detected by post mortem toxicology.

**Results:**

Toxicology reports were available for 13 541 of 14 206 people who died by suicide (95.3%; 10 246 men, 75.7%); poisoning with medicines contributed to 1163 deaths (8.6%). At least one PBS‐subsidised medicine had been dispensed around the time of death to 7998 people (59.1%). For three medicine classes, the proportions of people in whom the medicines were detected post mortem and their death was deemed medicine‐related were larger for those without records of recent dispensing than for people for whom they had been dispensed around the time of death: antidepressants (17.7% *v* 12.0%), anxiolytics (16.3% *v* 14.8%), and sedatives/hypnotics (24.3% *v* 16.5%). At least one recently dispensed medicine not detected post mortem was identified for 6208 people (45.8%).

**Conclusions:**

A considerable proportion of people who died by suicide were not taking psychotropic medicines recently dispensed to them, suggesting non‐adherence to pharmacotherapy, and a smaller than expected proportion were using antidepressants. Conversely, medicines that had not recently been dispensed were detected post mortem in many people for whom poisoning with medicines was a contributing factor, suggesting medicine stockpiling.

**The known**: The role of medicines in suicide is complex: they are a common means of suicide in high income countries, non‐adherence to psychotropic therapy is a challenge for health care professionals treating people at risk of suicide, and some medicines are themselves associated with increased risk of self‐harm.

**The new**: Mismatches between the dispensing of medicines around the time of death from suicide and post mortem toxicology findings suggest considerable levels of non‐adherence to psychotropic pharmacotherapy and inappropriate psychotropic medicine accumulation.

**The implications**: Monitoring adherence to psychotropic therapy, cessation of therapy, and safe storage and disposal of medicines are important for reducing the risk of suicide.

As mental illness^1^, ^2^ and many physical disorders are risk factors for suicide,^3^ the appropriate use of medicines for treating people with these conditions plays a role in suicide prevention. However, medicine overdose, especially with psychotropic medicines, is among the most common suicide methods in high income countries,^4^ and the incidence of non‐fatal deliberate self‐poisoning is also rising.^5^ Moreover, non‐adherence to psychotropic therapy is a key challenge for clinicians caring for people at risk of suicide,^6^ and some psychotropic and non‐psychotropic medicines have been associated with increased risk of suicidal behavior.^7^, ^8^, ^9^, ^10^

Medicine use at the time of death by suicide has been investigated by analysing administrative claims^11^, ^12^ and forensic toxicology data.^13^, ^14^ The former typically provide information on whether a medicine was dispensed, the latter on whether it was used. Studies applying these approaches, mostly in high income countries, have found similar rates of medicine use at the time of death, but have not linked sources of the two data types. Data linkage could provide a more robust overview of medicine use at the time of death and consequently of the medical conditions being treated at this point, improving our knowledge of therapy adherence and medicine use by people who die by suicide.

We therefore analysed linked whole population Australian data on medicine dispensing, toxicology, and mortality to determine the numbers and types of medicines dispensed around the time of death to people who died by suicide, and to compare the medicines listed in post mortem toxicology reports with those recently dispensed.

## Methods

We analysed data from the Australian Suicide Prevention using Health Linked Data (ASHLi) study, a population‐based case series study of closed coronial cases for deaths of people in Australia aged ten years or more during 1 July 2013 – 10 October 2019 (date of data extraction) deemed by coroners to be the result of intentional self‐harm.^15^ In the ASHLi study, National Coronial Information System (NCIS) data were linked with several administrative datasets, including that of the Pharmaceutical Benefits Scheme (PBS).

Any death in Australia suspected to have been a suicide is investigated by a coroner. The NCIS includes detailed information pertaining to each coronial inquiry, including the demographic characteristics of the deceased person, the details and circumstances of their death, and autopsy findings, including any toxicology findings. For this study, we examined toxicology report information regarding drugs and their metabolites detected at autopsy. Post mortem toxicology screening does not encompass all medications, but typically includes almost all psychotropic and analgesic medicines.

For our analysis, we included all PBS‐listed medicines dispensed in the community, by private hospitals, or (for most states) on discharge from public hospitals. The PBS does not subsidise non‐prescription, complementary and alternative medicines, or medicines dispensed within public hospitals to inpatients. Private prescriptions (ie, the patient pays the entire cost of the medicine) are not included in PBS data. The PBS captures the date the medicine was dispensed and medicine‐specific information, including the PBS item code, linked with the World Health Organization Anatomical Therapeutic Chemical (ATC) classification.^16^

### Data linkage

Coronial records for suicide deaths that matched our inclusion criteria were identified by the NCIS, which provided the relevant personal identifiers to the Australian Institute of Health and Welfare (AIHW) for probabilistic linkage with PBS records (and other administrational datasets not pertinent to this article). De‐identified linked data were provided to the researchers.

### Measures

We extracted data on age, sex, marital status, employment status, remoteness of residence at time of death (Statistical Area 3^17^), and method of death (as recorded in cause of death field: hanging, poisoning, falls, firearm, drowning, sharp object, moving object, or other) for each deceased person. Each record was then further classified according to whether the coroner found that the death involved poisoning with a medicine; that is, one or more medicines were found to have contributed to the death, regardless of whether they were the primary cause.

We categorised medicines by ATC code according to the anatomical main group (ATC first level), medicine class (ATC third level), and the specific medicine (ATC fifth level).

As the prescribed dose of medicines cannot be directly ascertained from PBS records, which include only information on the quantity and strength of medicines dispensed, we estimated medicine exposure time using the PBS 10% sample dataset, a standard dataset provided by Services Australia; it includes all prescription medicine claims for a nationally representative, random 10% sample of PBS‐eligible Australian residents.^16^ For each medicine, we calculated the number of days between two consecutive dispensings for each person in the PBS 10% sample dataset dispensed the medicine during the period covered by the ASHLi study; we defined the period of exposure as the number of days within which 75% of people in the PBS 10% sample dataset had received a second dispensing of the same medicine. For medicines not dispensed in sufficient quantity to reliably estimate the period of exposure, it was deemed to be fourteen days, as described in an earlier study.^18^ If the period between the final dispensing and the date of death was less than the estimated exposure period, the medicine was deemed to have been dispensed around the time of death.

### Comparison of recent dispensing and post mortem detection of medicines

We compared the recent dispensing and post mortem detection of specific medicines. For psychotropic medicines routinely screened for during autopsy (antidepressants, antipsychotics, anxiolytics, opioids, sedatives/hypnotics), we compared the medicine classes detected post mortem with those deemed to have been dispensed around the time of death, using three dispensing/detection categories: the medicine was dispensed and detected post mortem; the medicine was dispensed but not detected post mortem; and the medicine was detected but had not recently been dispensed.

### Statistical analysis

All analyses were conducted in R 4.2.1 (R Foundation for Statistical Computing). We calculated the proportions of people for whom medicines were dispensed around the time of death (case frequency), by medicine group, class, and specific medicine; we report the age and sex characteristics of people dispensed these medicines, and the proportion for whom poisoning with one or more medicines was implicated in their deaths. We also determined the number of different medicines dispensed to each person around the time of death.

For the most frequently dispensed psychotropic medicine classes, we determined the proportions of people for whom a medicine from that class was dispensed or detected in each dispensing/detection category. We then determined the proportion of any medicine‐related deaths among people dispensed each medicine class and detection/dispensing category.

### Ethics approval

Our study was approved by the Justice Human Research Ethics Committee of the Victorian Department of Justice and Community Safety (CF/17/23250), the Western Australian Coronial Ethics Committee (EC 14/18 M0400), the AIHW Ethics Committee (EO2017/4/366), and the NSW Population and Health Services Research Ethics Committee (2017/HRE1204, 2013/11/494). Access to the 10% PBS sample was granted by the Services Australia external request evaluation committee (RMS2076).

## Results

Of the 14 637 NCIS records for people who died by suicide, 14 354 could be linked by the AIHW to PBS records (98.1%); 148 were excluded after assessing the consistency of key data across the linked datasets. Of the remaining 14 206 cases, forensic toxicology examination reports were available for 13 541 people and were included in our analysis (95.3% of eligible linked record sets); their median age was 44 years (interquartile range [IQR], 31–57 years), and 10 246 were men (75.7%) (Box 1).

Box 1 **Characteristics of 13 541 people who died by suicide in Australia, 1 July 2013 – 10 October 2019**

**Table:** 

Characteristic	Number of people
Total number of deceased persons	13 541
Age (years), median (IQR)	44 (31–57)
10–24	1843 (13.6%)
25–44	5070 (37.4%)
45–64	4525 (33.4%)
65 or older	2103 (15.5%)
Sex (men)	10 246 (75.7%)
Geographic remoteness*	
Major cities	8274 (61.1%)
Inner regional	2960 (21.9%)
Outer regional	1453 (10.7%)
Remote/very remote	435 (3.2%)
Missing data	419 (3.1%)
Employment status	
Employed	4790 (35.4%)
Unemployed	3578 (26.4%)
Student	618 (4.6%)
Retired/pensioner	2579 (19.0%)
Other	173 (1.3%)
Missing data	1803 (13.3%)
Marital status	
Never married	3691 (27.2%)
Married	4666 (34.5%)
Separated/divorced	2777 (20.5%)
Widowed	563 (4.2%)
Missing data	1844 (13.6%)
Method of death^†^	
Hanging	7422 (54.8%)
Any poisoning	3366 (24.9%)
Poisoning with medicines^‡^	1163 (8.6%)
Falls	668 (4.9%)
Firearm	771 (5.7%)
Sharp object	399 (2.9%)
Moving object	586 (4.3%)
Other	750 (5.5%)

IQR = interquartile range.

* Australian Statistical Geography Standard (ASGS).^17^

† Multiple causes possible.

‡ In the National Coronial Information System records, a further 837 deaths (6.2%) under “any poisoning” had “mixed drug” listed as a contributor to death, without specifying the drugs involved.

### Medicines dispensed around the time of death

At least one PBS‐subsidised medicine had been dispensed around the time of death to 7998 people (59.1%); their median age was 49 years (IQR, 36–63 years), and 5620 were men (70.3%). Two or more medicines had been dispensed around the time of death to 5637 people (41.6%) and five or more to 1943 (14.3%). The most frequently dispensed medicine classes, both overall (6456 of 13 541, 47.7%) and for each combination of sex and age group were nervous system medicines (Box 2, Box 3). The most frequently dispensed specific medicines were the nervous system medicines diazepam, mirtazapine, temazepam, quetiapine, and escitalopram (Box 4).

Box 2 Dispensing of medicines around the time of death to 13 541 people who died by suicide in Australia, 1 July 2013 – 10 October 2019, by Anatomical Therapeutic Classification (ATC) first level category, sex, and age group*

**Figure:**
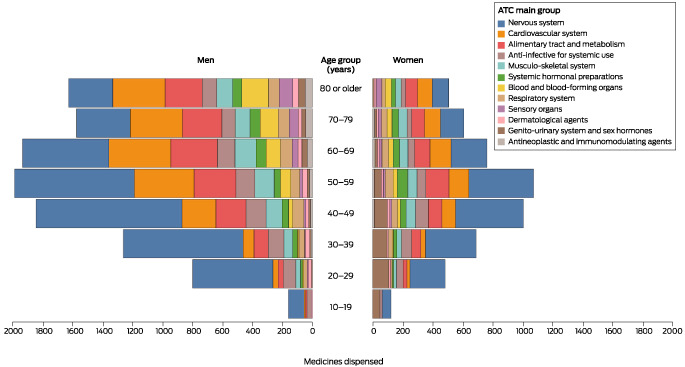
* Data for medicines dispensed to fewer than six people by age/sex group are not included in the figure.

Box 3 **The twenty medicine classes (Anatomical Therapeutic Classification [ATC] third level) most frequently dispensed around the time of death to 13 541 people who died by suicide in Australia, 1 July 2013 – 10 October 2019**

**Table:** 

Medicine class (ATC third level)	Dispensing frequency*	Case frequency^†^	Median age, years (IQR)	Men	Poisoning with medicines^‡^
Antidepressants (N06A)	7078	4183 (30.9%)	47 (35–59)	2748 [65.7%]	497 [11.9%]
Anxiolytics (N05B)	3423	1810 (13.4%)	47 (36–57)	1140 [63.0%]	260 [14.4%]
Antipsychotics (N05A)	2816	1525 (11.3%)	44 (33–55)	1012 [66.4%]	199 [13.0%]
Opioids (N02A)	3006	1359 (10.0%)	53 (41–68)	880 [64.8%]	266 [19.6%]
Drugs for peptic ulcer/gastro‐oesophageal reflux disease (A02B)	1873	1345 (9.9%)	62 (50–75)	953 [70.9%]	218 [16.2%]
Lipid‐modifying agents, plain (C10A)	1314	1023 (7.6%)	66 (56–78)	799 [78.1%]	145 [14.2%]
Hypnotics/sedatives (N05C)	1558	978 (7.2%)	55 (42–68)	630 [64.4%]	129 [13.2%]
Other analgesics and antipyretics (N02B)	1256	725 (5.4%)	60 (46–74)	475 [65.5%]	142 [19.6%]
Beta‐blocking agents (C07A)	913	641 (4.7%)	63 (50–77)	480 [74.9%]	112 [17.5%]
Anti‐inflammatory/anti‐rheumatic agents, non‐steroid (M01A)	765	576 (4.3%)	54 (42–66)	387 [67.2%]	83 [14%]
Adrenergic agents, inhalants (R03A)	1175	564 (4.2%)	59 (44–72)	359 [63.7%]	95 [17%]
Anti‐thrombotic agents (B01A)	830	555 (4.1%)	73 (61–82)	444 [80.0%]	82 [15%]
Beta‐lactam antibacterial agents, penicillins (J01C)	581	482 (3.6%)	49 (37–67)	338 [70.1%]	54 [11%]
Angiotensin‐converting enzyme inhibitors, plain (C09A)	583	473 (3.5%)	66 (56–78)	398 [84.1%]	62 [13%]
Anti‐epileptics (N03A)	764	461 (3.4%)	48 (38–59)	266 [57.7%]	75 [16%]
Angiotensin II receptor blockers, plain (C09C)	530	437 (3.2%)	66 (55–78)	319 [73.0%]	58 [13%]
Blood glucose‐lowering agents, excl. insulins (A10B)	732	405 (3.0%)	62 (52–71)	322 [79.5%]	63 [16%]
Corticosteroids for systemic use, plain (H02A)	506	348 (2.6%)	65 (48–76)	224 [64.4%]	52 [15%]
Angiotensin II receptor blockers, combinations (C09D)	400	333 (2.5%)	63 (54–73)	261 [78.4%]	32 [10%]
Hormonal contraceptives for systemic use (G03A)	379	325 (2.4%)	30 (22–39)	—	39 [12%]

IQR = interquartile range.

* Dispensing of medicines in class for which estimated duration of therapy included the date of death.

† Individuals dispensed at least one medicine from the class, and estimated duration of therapy included date of death.

‡ Individuals dispensed at least one medicine from the class, and poisoning with any medicine was deemed to have contributed to their death.

Box 4 **The twenty medicines most frequently dispensed around the time of death to 13 541 people who died by suicide in Australia, 1 July 2013 – 10 October 2019**

**Table:** 

Medicine	Dispensing frequency*	Case frequency^†^	Median age, years (IQR)	Men	Poisoning with medicines^‡^
Diazepam	2687	1466 (10.8%)	45 (35–55)	939 [64.1%]	197 [13%]
Mirtazapine	1203	871 (6.4%)	52 (40–63)	607 [69.7%]	81 [9%]
Temazepam	1325	861 (6.4%)	54 (41–68)	563 [65.4%]	102 [12%]
Quetiapine	1176	673 (5.0%)	43 (33–55)	390 [57.9%]	124 [18%]
Escitalopram	807	588 (4.3%)	44 (33–55)	410 [69.7%]	44 [8%]
Esomeprazole	769	562 (4.2%)	60 (48–72)	371 [66.0%]	99 [18%]
Venlafaxine	871	555 (4.1%)	47 (37–59)	335 [60.4%]	71 [13%]
Oxycodone	953	494 (3.6%)	56 (42–70)	323 [65.4%]	114 [23%]
Atorvastatin	602	479 (3.5%)	67 (55–79)	391 [81.6%]	63 [13%]
Codeine/paracetamol	708	478 (3.5%)	47 (37–59)	303 [63.4%]	76 [16%]
Sertraline	673	477 (3.5%)	46 (32–59)	311 [65.2%]	52 [11%]
Pantoprazole	603	469 (3.5%)	63 (51–75)	344 [73.3%]	72 [15%]
Desvenlafaxine	665	468 (3.5%)	46 (35–55)	320 [68.4%]	32 [7%]
Pregabalin	748	430 (3.2%)	51 (41–64)	275 [64.0%]	91 [21%]
Olanzapine	613	415 (3.1%)	47 (36–58)	305 [73.5%]	37 [9%]
Amitriptyline	713	394 (2.9%)	52 (42–63)	222 [56.3%]	97 [25%]
Paracetamol	508	353 (2.6%)	70 (57–81)	230 [65.2%]	61 [17%]
Rosuvastatin	412	336 (2.5%)	64 (54–75)	248 [73.8%]	47 [14%]
Fluoxetine	481	332 (2.5%)	44 (28–55)	190 [57.2%]	46 [14%]
Oxazepam	518	310 (2.3%)	55 (44–68)	177 [57.1%]	62 [20%]

IQR = interquartile range.

* Dispensing of medicines in class for which estimated duration of therapy included the date of death; multiple dispensing to individuals is possible.

† Individuals dispensed at least one medicine from the class, and estimated duration of therapy included date of death.

‡ Individuals dispensed medicines for whom poisoning with any medicine was deemed to have contributed to their death.

### Comparison of medicines dispensed and detected post mortem

Medicines (or their metabolites) were detected post mortem in 9042 people (66.8%). At least one medicine not dispensed around the time of death was detected in 7135 people (52.7%) (Box 5), for 1042 of whom (14.6%) poisoning with one or more medicines was implicated in their deaths.

Box 5 **The twenty medicines most frequently detected post mortem but not dispensed around the time of death for 13 541 people who died by suicide in Australia, 1 July 2013 – 10 October 2019**

**Table:** 

	Number of people		
Medicine/metabolites	Medicine/metabolites detected	Medicine dispensed outside exposure period	Comments
Paracetamol/metabolites	1377	478 (34.7%)	Available without prescription
Diazepam/metabolites	1051	571 (54.3%)	Often prescribed pro re nata
Morphine/metabolites	862	27 (3.1%)	Administered before or during hospitalisation for pain; can be detected as metabolite of codeine or heroin
Temazepam	820	268 (32.7%)	Often prescribed pro re nata; can also be detected as metabolite of diazepam
Oxazepam	778	106 (13.6%)	Often prescribed pro re nata, can also be detected as metabolite of diazepam
Codeine/metabolites	659	245 (37.2%)	Available without prescription until February 2018
Doxylamine/metabolites	542	< 6	Available without prescription, not subsidised by PBS
Ibuprofen/metabolites	511	22 (4.3%)	Available without prescription
Nortriptyline/metabolites	474	15 (3.2%)	Can be detected as metabolite of amitriptyline
Midazolam	332	< 6	Almost exclusively administered in hospital or by paramedics to people with seizures, agitation
Quetiapine	302	144 (47.7%)	Used for many off‐label indications not subsidised by the PBS
Metoclopramide	301	98 (33%)	Used in hospitals as an anti‐emetic; available without prescription
Oxycodone	277	140 (50.5%)	
Clonazepam/metabolites	261	17 (6.5%)	Often prescribed pro re nata
Mirtazapine	221	147 (66.5%)	Often prescribed privately
Phenobarbitone	216	< 6	
Promethazine	199	< 6	Available without prescription
Olanzapine	199	103 (51.8%)	
Quinine	183	< 6	
Metoprolol	183	< 6	

PBS = Pharmaceutical Benefits Scheme.

* A comprehensive list of medicines detected post mortem but not dispensed around the time of death is included in the Supporting Information, table 1.

For three of the most frequently dispensed nervous system medicine classes, the proportions of people in whom the medicines were detected post mortem and their death was deemed medicine‐related were larger for those without records of recent dispensing than for people for whom they had been dispensed around the time of death: antidepressants (17.7% *v* 12.0%), anxiolytics (16.3% *v* 14.8%), and sedatives/hypnotics (24.3% *v* 16.5%) (Box 6).

Box 6 **Nervous system medicine classes dispensed to or detected post mortem in 13 541 people who died by suicide in Australia, 1 July 2013 – 10 October 2019**

**Table:** 

Medicine class (ATC third level class)	Frequency* (proportion of people)	Poisoning with medicines^†^
**Antidepressant (N06A)**		
Dispensed or detected^‡^	5152 (38.0%)	634 (12.3%)
Dispensed and detected	3523 (26.0%)	423 (12.0%)
Detected, not dispensed^§^	1534 (11.3%)	272 (17.7%)
Dispensed, not detected	890 (6.6%)	98 (11%)
**Antipsychotic (N05A)**		
Dispensed or detected^‡^	2172 (16.0%)	285 (13.1%)
Dispensed and detected	1021 (7.5%)	150 (14.7%)
Detected, not dispensed^§^	786 (5.8%)	110 (14.0%)
Dispensed, not detected	611 (4.5%)	71 (12%)
**Anxiolytic (N05B)**		
Dispensed or detected^‡^	3453 (25.5%)	525 (15.2%)
Dispensed and detected	1569 (11.6%)	232 (14.8%)
Detected, not dispensed^§^	2098 (15.5%)	342 (16.3%)
Dispensed, not detected	299 (2.2%)	41 (14%)
**Opioids (N02C)**		
Dispensed or detected^‡^	2878 (21.3%)	558 (19.4%)
Dispensed and detected	848 (6.3%)	203 (23.9%)
Detected, not dispensed^§^	1928 (14.2%)	393 (20.4%)
Dispensed, not detected	671 (5.0%)	96 (14%)
**Sedatives/hypnotics (N05C)**		
Dispensed or detected^‡^	2719 (20.1%)	552 (20.3%)
Dispensed and detected	629 (4.6%)	104 (16.5%)
Detected, not dispensed^§^	1840 (13.6%)	447 (24.3%)
Dispensed, not detected	359 (2.7%)	26 (7.2%)

* Unique individuals dispensed the medicine around time of death or in whom medicine was detected post mortem; multiple categories within drug class possible.

† Proportion of unique individuals to whom the medicine was dispensed or in whom it was detected post mortem for whom poisoning with any medicines contributed to death.

‡ As several medicines may have been dispensed from the same class or detected post mortem for an individual, the value for this category is not the sum of the following two categories.

§ Dispensing records for detected medicines during the year before death but not during the estimated period of exposure were: antidepressant, 825 (53.8%); antipsychotic, 339 (43.1%); anxiolytic, 700 (33.4%); opioid, 495 (25.7%); and sedative, 307 (16.7%).

At least one medicine dispensed around the time of death but not detected post mortem was identified for 6208 people (45.8%); the most frequently dispensed but not detected (of those routinely screened for post mortem) was temazepam (326 people, 2.4%) (Supporting Information, table 2).

## Discussion

We found that PBS‐subsidised medicines had been recently dispensed to 59% of people who died by suicide in Australia during 2013–2019, and that more than one medicine had been dispensed to 42%. The most frequently dispensed drugs were nervous system medicines, and the most frequent class dispensed was antidepressants. One or more medicines not recently dispensed were detected post mortem in 53% of people who died by suicide.

The proportions of people in our study dispensed medicines from most classes were larger than for the overall Australian population in 2018; the differences were particularly marked for nervous system medicines (antidepressants: 30.9% *v* 7.4%; antipsychotics: 11.3% *v* 1.4%; anxiolytics: 13.4% *v* < 1%; opioids: 10.0% *v* 2.6%; hypnotics/sedatives: 7.2% *v* < 1%)^18^ (Supporting Information, table 3). These differences are partly attributable to differences in age and sex distributions, but the generally higher dispensing rates for most medicines probably reflect the chronic nature of physical and mental disorders in people who die by suicide.

We found that antidepressants had recently been dispensed to or were detected post mortem in 38% of people who died by suicide, a smaller proportion than expected given their burden of depression and anxiety; the Global Burden of Disease Study 2010 estimated that depression is responsible for 46.1% (95% confidence interval [CI], 28.0–60.8%) of the disability‐adjusted life years (DALYs) lost to suicide, and anxiety disorders for 7.4% (95% CI, 3.0–12.7%).^2^ Our finding suggests that many people with depression or anxiety were not adhering to prescribed pharmacotherapy, were not being treated with antidepressants, or were not being treated for depression or anxiety around the time of their deaths. Indeed, another Australian data linkage study found low levels of mental health service use prior to suicide,^19^ and the estimated mental health treatment rate for people with mental illness in Australia in 2014 was only 35%.^20^ Our finding may therefore reflect generally low levels of help‐seeking by people with mental illness.

Antipsychotics had been recently dispensed to or were detected post mortem in 16% of people, more consistent with the numbers of suicide‐related lost DALYs attributed to bipolar disorder (5.4%; 95% CI, 1.8–10.7%) and schizophrenia (4.7%; 95% CI, 4.1–5.3%).^2^ We have no information on the indications for medicine prescribing, but our findings suggest that people with schizophrenia or bipolar disorder were more frequently using pharmacotherapy at the time of death than those with depression or anxiety.

Many medicines dispensed around the time of death were not detected post mortem (Supporting Information, table 2), suggesting non‐adherence to therapy. As cessation of antidepressants^21^ and opioids^22^ has been associated with greater risk of suicide, the possibility of non‐adherence and the importance of monitoring medicine use^6^ and assisting with safe tapering and cessation of medicines^23^ should be borne in mind.

In our study, poisoning by medicines was identified as a contributor to 8.6% of deaths. However, the proportions of medicine poisoning‐related deaths were larger for people recently dispensed most medicine types, and was largest for those dispensed opioids (19.6%) (Box 3). This finding indicates that dispensing of prescription medicines provides access to means for self‐harm, reinforcing the need for caution when prescribing toxic medicines for patients at risk of suicide.^13^ Further, these medicines may be taken in combination with other medicines or substances, leading to synergistic toxic effects.^24^

We also found that the proportions of deaths in which poisoning with medicine was implicated were larger for people in whom antidepressants, anxiolytics, or sedatives/hypnotics were detected but had not been dispensed around the time of death than for those for whom the medicine had been dispensed around the time of death (Box 6). This finding suggests that some medicines likely to have been used for suicide were obtained on private prescriptions, had not been dispensed to the person who died (ie, were obtained illegally or from another household member), or had been dispensed many months before the death of the person who used them. The latter possibility may reflect medicine stockpiling or unintentional accumulation, each of which increases access to means for suicide and highlights the importance of safe disposal of unused medicines.

### Limitations

Our study overcame many limitations of analysing coronial data or dispensing data alone by examining medicine use according to whether it was dispensed and used by individuals around the time of their deaths. We have nevertheless probably underestimated medicine use. The PBS does not subsidise all dispensed medicines, and post mortem toxicology does not routinely screen for all medicines. Moreover, several factors influence post mortem detectability of drugs, including technological limits of detection and reporting,^25^ post mortem changes,^26^ and drug stability.^27^ As we did not assess combinations of medicines with other medicines or with substances such as alcohol and illicit drugs, we cannot draw conclusions about synergistic toxicity related to drug–drug interactions. The time between death, finalisation of the coronial enquiry, and data linkage means that we did not include all suicide deaths, particularly during the latter years of the series.^15^

### Conclusions

We found that the proportion of people who died by suicide during 2013–19 who were using antidepressants at the time of their deaths was lower than would be expected given the estimated burden of depression and anxiety in people who die by suicide,^2^ perhaps reflecting suboptimal levels of treatment for people with mental illness. A considerable number of people were dispensed psychotropic medicines in the period preceding their death that were not detected post mortem, suggesting non‐adherence to treatment. Finally, medicines were detected post mortem in many people for whom they had not recently been dispensed, indicating the need to curb the inappropriate intentional or unintentional stockpiling of medicines.

## Open access

Open access publishing facilitated by The University of Sydney, as part of the Wiley – The University of Sydney agreement via the Council of Australian University Librarians.

## Competing interests

No relevant disclosures.

If you or anyone you know is experiencing distress, please call Lifeline on 13 11 14 (www.lifeline.org.au) or Beyond Blue (www.beyondblue.org.au) on 1300 22 46 36.

## Supporting information


Supplementary results
