# Supplementary material for: Psychotropic and other medicine use at time of death by suicide: a population‐level analysis of linked dispensing and forensic toxicology data
Source: Med J Aust. 2023 May 25;219(2):63–9. doi: 10.5694/mja2.51985 (PMC10952140; doi:10.5694/mja2.51985)
Supplement: Supplementary file 1 — Supplementary results [file MJA2-219-63-s001.pdf]

The Medical Journal of Australia

Supporting Information

Supplementary results

This appendix was part of the submitted manuscript and has been peer reviewed. It is posted as supplied by the authors.

Appendix to: Chitty KM, Buckley NA, Lim J, et al. Psychotropic and other medicine use at time of death by suicide: a population-level analysis of linked dispensing and forensic toxicology data. *Med J Aust* 2023; doi: 10.5694/mja2.51985.

Table 1. Medicines detected post mortem but not captured in an individual's proximal to death dispensing records, and proportions of people dispensed the medicines outside the estimated periods of exposure (ie, not proximal to death) (medicines with fewer than six detections not shown)

Medicine	People in whom the medicine was detected	People for whom medicine was dispensed outside the exposure period
Paracetamol	1375	476 (34.6%)
Temazepam	820	268 (32.7%)
Oxazepam	778	106 (13.6%)
Diazepam	559	312 (55.8%)
Doxylamine	529	<6
Morphine (free)	455	13 (2.9%)
Nordiazepam	454	241 (53.1%)
Nortriptyline	450	14 (3.1%)
Codeine (free)	446	161 (36.1%)
Ibuprofen	445	19 (4.3%)
Midazolam	332	<6
Quetiapine	302	144 (47.7%)
Metoclopramide	301	98 (33%)
Oxycodone	277	140 (50.5%)
Mirtazapine	221	147 (66.5%)
Pentobarbitone	216	<6
7-aminoclonazepam	214	13 (6.1%)
Promethazine	199	<6
Olanzapine	199	103 (51.8%)
Morphine (total)	197	9 (5%)
Metoprolol	183	<6
Quinine	183	<6
Citalopram	182	130 (71.4%)
Alprazolam	177	43 (24%)
Salicylic acid	174	<6
Zolpidem	169	<6
Lidocaine	167	<6
Zopiclone	165	<6
Amitriptyline	153	74 (48%)
Methadone	149	7 (5%)
Ketamine	145	<6
Morphine-3-glucuronide	138	<6
Tramadol	129	57 (44%)
Lignocaine	121	<6
Lorazepam	118	<6
Sertraline	117	75 (64%)
Codeine (total)	96	35 (36%)
Codeine-6-glucuronide	95	44 (46%)
Pholcodine	92	<6
Fentanyl	84	11 (13%)
Nicotine	74	<6
Fluoxetine	71	51 (72%)
Lamotrigine	71	19 (27%)
Propofol	68	<6

Medicine	People in whom the medicine was detected	People for whom medicine was dispensed outside the exposure period
Cotinine	67	<6
Ibuprofen metabolite	66	<6
Desmethylvenlafaxine	66	53 (80%)
Opioid, unspecified	63	<6
Venlafaxine	63	48 (76%)
Pseudoephedrine	61	<6
Duloxetine	61	47 (77%)
Levetiracetam	58	<6
Valproic acid	58	38 (66%)
Amiodarone	54	<6
Metformin	53	40 (76%)
7-Aminonitrazepam	52	27 (52%)
Naloxone	52	19 (36%)
Diphenhydramine	51	<6
Atropine	50	<6
Zuclopenthixol	49	<6
Amlodipine	48	35 (73%)
Clonazepam	47	<6
Telmisartan	46	36 (78%)
Ondansetron	45	<6
Pregabalin	45	33 (73%)
Naproxen	44	17 (39%)
Morphine-6-glucuronide	42	<6
Sildenafil	42	<6
Propranolol	42	28 (67%)
Ephedrine	39	<6
Irbesartan	38	31 (82%)
Norfluoxetine	37	31 (84%)
Desmethyldiazepam	37	17 (46%)
Phenytoin	36	<6
Desvenlafaxine	36	27 (75%)
Paliperidone	36	24 (67%)
Phentermine	34	<6
Aripiprazole	34	23 (68%)
O-Desmethylvenlafaxine	33	26 (79%)
Dextromethorphan	29	<6
Hydrocodone	29	<6
6-monoacetylmorphine	29	<6
Levamisole	28	<6
Norbuprenorphine	26	<6
Atenolol	26	22 (85%)
Warfarin	26	16 (62%)
Noroxycodone	26	15 (58%)
Carbamazepine	26	13 (50%)
Pantoprazole	25	<6
Nitrazepam	25	11 (44%)
Nortriptyline metabolite	24	<6

Medicine	People in whom the medicine was detected	People for whom medicine was dispensed outside the exposure period
Dextropropoxyphene	23	<6
Haloperidol	23	<6
Fluvoxamine	23	19 (83%)
Quetiapine metabolite	22	9 (41%)
Ranitidine	21	<6
Fluconazole	21	<6
Paroxetine	21	19 (90%)
O-desmethyltramadol	21	12 (57%)
Hydromorphone	20	<6
Tadalafil	19	<6
Clozapine	19	6 (32%)
Trimethoprim	18	9 (50%)
Buprenorphine	17	<6
Codeine metabolite	17	<6
Citalopram metabolite	17	12 (71%)
Mirtazapine metabolite	17	9 (53%)
Bromazepam	16	<6
Prochlorperazine	16	<6
Amphetamine	16	16 (100%)
Gliclazide	16	12 (75%)
Celecoxib	16	11 (69%)
Desmethylcitalopram	16	11 (69%)
Meloxicam	16	10 (62%)
Desmethylsertraline	15	11 (73%)
Hydroxyrisperidone	15	10 (67%)
Metronidazole	14	<6
Atorvastatin	14	11 (79%)
Clomipramine	14	8 (57%)
Doxylamine metabolite	13	<6
Pheniramine	13	<6
Risperidone	13	9 (69%)
Lithium	13	8 (62%)
Diltiazem	13	7 (54%)
Doxepin	13	7 (54%)
Verapamil	12	<6
Diclofenac	12	<6
Hydroxychloroquine	12	7 (58%)
Chloroquine	11	<6
Glucose	11	<6
Omeprazole	11	<6
Amisulpride	11	<6
Chlorpromazine	11	<6
Flunitrazepam	10	<6
Midazolam metabolite	10	<6
Modafinil	10	<6

Table 2. Medicines dispensed around the time of death but not detected post mortem (medicines with fewer than ten detections not shown)

Medicine	Frequency
Esomeprazole	564
Atorvastatin	473
Pantoprazole**	441
Amoxicillin**	436
Perindopril	395
Paracetamol*	374
Rosuvastatin	335
Temazepam*	326
Pregabalin**	325
Salbutamol**	279
Cefalexin	277
Hydrochlorothiazide**	270
Codeine*	254
Furosemide (frusemide)	240
Potassium chloride	237
Metformin**	232
Amlodipine**	228
Levothyroxine	224
Prednisolone**	224
Oxycodone*	223
Fluticasone propionate	221
Aspirin	218
Clavulanic acid	207
Salmeterol**	204
Quetiapine*	199
Bicarbonate	193
Macrogol-3350	192
Metoprolol tartrate	184
Diazepam*	181
Sodium chloride	179
Budesonide	173
Formoterol (eformoterol)	172
Venlafaxine*	164
Allopurinol	160
Ramipril	157
Simvastatin	156
Candesartan	155
Meloxicam**	155
Tiotropium	152
Etonogestrel	151
Ethinylestradiol	147
Naloxone	147
Rabeprazole**	146
Risperidone*	146
Levonorgestrel	142
Lithium	138
Valproate	136
Mirtazapine*	134
Celecoxib**	130
Olanzapine*	122
Citalopram*	116
Omeprazole	116
Clopidogrel**	108
Doxycycline	108
Irbesartan**	103
Telmisartan**	101
Oxazepam*	96
Spironolactone	94
Denosumab	89
Amitriptyline*	84
Hydroxocobalamin	84
Lercanidipine**	84
Ezetimibe	82
Prazosin**	82
Timolol*	82
Betamethasone dipropionate	79
Indapamide**	78
Tramadol*	78
Duloxetine*	77
Insulin glargine	76
Levodopa	76
Rivaroxaban	75
Diclofenac**	72
Dosulepin (dothiepin)*	72
Propranolol*	71
Baclofen**	70
Estradiol	70
Dutasteride	69
Mometasone	69
Buprenorphine	67
Latanoprost	67
Metoclopramide*	67
Tamsulosin	67
Prochlorperazine	66
Valsartan**	65
Dexamethasone	64
Atenolol*	63
Insulin aspart	63
Prednisone	59
Clonidine	57
Triamcinolone	56
Bisoprolol**	55

Medicine	Frequency
Olmesartan	55
Nicotine*	54
Olmesartan medoxomil	54
Digoxin	53
Valaciclovir	53
Domperidone	52
Glyceryl trinitrate	51
Sertraline*	51
Fenofibrate	50
Carbidopa	49
Isosorbide mononitrate	49
Nystatin	47
Betamethasone valerate	46
Apixaban	45
Folic acid	45
Indometacin	44
Ranitidine**	44
Trimethoprim**	44
Gliclazide**	42
Gramicidin	42
Oxybutynin**	41
Warfarin**	41
Bimatoprost	39
Felodipine**	39
Naproxen**	38
Nitrazepam*	38
Sitagliptin**	38
Moxonidine	37
Paroxetine*	37
Carmellose sodium	36
Ibuprofen**	36
Pramipexole	36
Emtricitabine	35
Benserazide	34
Risedronate	34
Zuclopenthixol decanoate	34
Acamprosate	33
Lansoprazole**	33
Neomycin	33
Medroxyprogesterone	32
Sulfamethoxazole**	32
Tenofovir disoproxil	32
Methotrexate**	31
Alendronate	30
Brimonidine	29
Fentanyl*	29
Morphine*	29
Varenicline	29

Medicine	Frequency
Alprazolam*	28
Chlorpromazine*	28
Hypromellose	28
Calcipotriol	27
Carvedilol**	27
Fluoxetine*	27
Indacaterol	27
Methylprednisolone**	27
Sumatriptan	27
Testosterone undecanoate	27
Zoledronic acid	27
Calcium carbonate	26
Goserelin	26
Vilanterol	26
Flucloxacillin**	25
Ipratropium**	25
Nifedipine*	25
Polyethylene glycol-400	25
Pravastatin	25
Propylene glycol	25
Benzatropine	24
Colchicine*	24
Pancreatic extract	24
Roxithromycin	24
Tapentadol	24
Asenapine	23
Colecalciferol	23
Glycopyrronium	23
Topiramate**	23
Clarithromycin	22
Naltrexone	22
Norethisterone	22
Periciazine	22
Sulfasalazine	22
Brinzolamide	21
Enalapril	21
Enoxaparin sodium	21
Loperamide	21
Minocycline	21
Travoprost	21
Dexamphetamine	20
Dextran-70	20
Metronidazole**	20
Nicorandil	20
Ondansetron	20
Terbutaline**	20
Carbimazole	19
Estriol	19

Medicine	Frequency
Famciclovir	19
Nitrofurantoin	19
Rizatriptan	19
Atropine sulfate	18
Ferrous fumarate	18
Haloperidol*	18
Diphenoxylate	17
Gabapentin**	17
Insulin lispro	17
Lactulose	17
Methylphenidate	17
Ticagrelor	17
Ziprasidone*	17
Bicalutamide	16
Ciprofloxacin	16
Dabigatran	16
Linagliptin	16
Lisinopril	16
Phenylephrine	16
Prednisolone acetate	16
Ranibizumab	16
Ciclesonide	15
Donepezil	15
Fluvoxamine*	15
Glimepiride**	15
Empagliflozin	14
Lamivudine	14
Pizotifen	14
Bisacodyl	13
Cefaclor	13
Dorzolamide	13
Entacapone	13
Fosinopril	13
Insulin isophane human	13
Levetiracetam**	13
Metoprolol succinate	13
Abacavir	12

Medicine	Frequency
Aflibercept	12
Calcitriol	12
Carbamazepine*	12
Chloramphenicol	12
Clindamycin	12
Dicloxacillin**	12
Dolutegravir	12
Fludrocortisone acetate	12
Framycetin sulfate	12
Isotretinoin	12
Leuprorelin	12
Mesalazine	12
Methenamine hippurate	12
Nizatidine**	12
Norethisterone acetate	12
Palonosetron	12
Umeclidinium	12
Botulinum toxin type a	11
Carbomer-980	11
Cobicistat	11
Fluorometholone	11
Fluorouracil	11
Fluticasone furoate	11
Hydralazine	11
Nebivolol	11
Aclidinium	10
Adapalene	10
Azithromycin	10
Benzoyl peroxide	10
Dipyridamole**	10
Elvitegravir	10
Fluorometholone acetate	10
Hydrocortisone	10
Lurasidone	10
Olodaterol	10
Thiamine	10
Vildagliptin	10

* Medicines routinely screened for in 2016 by the Victorian Institute of Forensic Medicine.

** Medicines routinely screened for during an acidic screen in 2016 by the Victorian Institute of Forensic Medicine. The basic, neutral, and acidic drug screen is only conducted when the case circumstances suggest use of corresponding drug types.

Other medicines are screened for only on specific request. However, the drugs routinely screened for is subject to changes over time and between jurisdictions.

Table 3. The twenty most frequently dispensed medicine classes around the time of death (in our study) and their prevalence in the Australian population on a typical day in 2018 (not age- or /sex-standardised)

Medicine class (ATC third level class)	Frequency of use at time of death*	Prevalence in Australian general population†
Antidepressants (N06A)	4184 (30.9%)	1,856,870 (7.4%)
Anxiolytics (N05B)	1811 (13.4%)	<1%
Antipsychotics (N05A)	1525 (11.3%)	338,290 (1.4%)
Opioids (N02A)	1359 (10.0%)	645,110 (2.6%)
Drugs for peptic ulcer/gastro-oesophageal reflux disease (A02B)	1345 (9.9%)	1,643,111 (6.6%)
Lipid-modifying agents (C10A,B)‡	1093 (8.1%)	2,219,200 (8.9%)
Hypnotics/sedatives (N05C)	981 (7.2%)	<1%
Other analgesics and antipyretics (N02B)	725 (5.4%)	378,680 (1.5%)
Beta-blocking agents (C07A)	641 (4.7%)	865,680 (3.5%)
Anti-inflammatory/anti-rheumatic agents, non-steroid (M01A)	576 (4.3%)	536,750 (2.1%)
Adrenergic agents, inhalants (R03A)	564 (4.2%)	765,666 (3.1%)
Anti-thrombotic agents (B01A)	555 (4.1%)	691,420 (2.8%)
Beta-lactam antibacterial agents, penicillins (J01C)	482 (3.6%)	552,370 (2.2%)
Angiotensin-converting enzyme inhibitors (C09A,B)‡	627 (4.6%)	1,090,460 (4.4%)
Anti-epileptics (N03A)	461 (3.4%)	483,334 (1.9%)
Angiotensin-converting enzyme inhibitors (C09C,D)‡	770 (5.7%)	1,502,340 (6.0%)
Blood glucose-lowering agents, excl. insulins (A10B)	405 (3.0%)	784,670 (3.1%)
Corticosteroids for systemic use, plain (H02A)	348 (2.6%)	336,950 (1.3%)
Hormonal contraceptives for systemic use (G03A)	325 (2.4%)	910,720 (3.6%)

* Individuals who were dispensed at least one medicine from the class, and the estimated duration of therapy included their date of death.

† Based on a typical day in Australian dispensing in 2018, estimated using Pharmaceutical Benefits Scheme data; the Australian population in 2018 was 24,992,370. Source: Wylie CE, Daniels B, Brett J et al. A national study on prescribed medicine use in Australia on a typical day. Pharmacoepidemiol Drug Saf 2020; 29: 1046-1053.

‡ To facilitate comparisons with the general population, the totals reported in this table include both plain and combination products.
